# Top-down Fabrication and Enhanced Active Area Electronic Characteristics of Amorphous Oxide Nanoribbons for Flexible Electronics

**DOI:** 10.1038/s41598-017-06040-2

**Published:** 2017-07-18

**Authors:** Hyun-June Jang, Ki Joong Lee, Kwang-Won Jo, Howard E. Katz, Won-Ju Cho, Yong-Beom Shin

**Affiliations:** 1 Department of Materials Science and Engineering, Johns Hopkins University, 3400 N Charles St, Baltimore, USA; 2 Hazards Monitoring BioNano Research Center, Korea Research Institute of Bioscience and Biotechnology, 125 Gwahak-Ro, Yuseong-Gu, Daejeon, 305-806 South Korea; 3 Department of Electronic Materials Engineering, Kwangwoon University, 20 Gwangun-ro, Nowon-gu, Seoul, 139-701 South Korea

## Abstract

Inorganic amorphous oxide semiconductor (AOS) materials such as amorphous InGaZnO (a-IGZO) possess mechanical flexibility and outstanding electrical properties, and have generated great interest for use in flexible and transparent electronic devices. In the past, however, AOS devices required higher activation energies, and hence higher processing temperatures, than organic ones to neutralize defects. It is well known that one-dimensional nanowires tend to have better carrier mobility and mechanical strength along with fewer defects than the corresponding two-dimensional films, but until now it has been difficult, costly, and impractical to fabricate such nanowires in proper alignments by either “bottom-up” growth techniques or by “top-down” e-beam lithography. Here we show a top-down, cost-effective, and scalable approach for the fabrication of parallel, laterally oriented AOS nanoribbons based on lift-off and nano-imprinting. High mobility (132 cm^2^/Vs), electrical stability, and transparency are obtained in a-IGZO nanoribbons, compared to the planar films of the same a-IGZO semiconductor.

## Introduction

Flexible and transparent electronics can be designed to maintain close contact with curved and moving surfaces, and can be inconspicuously mounted on a wide variety of substrates. Hence, many types of flexible/transparent electronic devices, including sensors^1–4^, thermoelectric devices^5, 6^, and displays^7, 8^, could be incorporated into everyday life in unobtrusive configurations^9^. Interest in these electronic devices has been growing rapidly over the past few years^10, 11^. So far, most of the technical issues impeding their fabrication are related to the need for low processing temperatures^12^. This is because most flexible materials, in particular substrates such as a plastic foil^13^ or paper^14^ sheet, are vulnerable to the high temperatures that are generally necessary to achieve satisfactory semiconductor property. The desirability of low processing temperatures has motivated the use of organic semiconductors, typically deposited and annealed below 150 °C. While their electrical performance is competitive to that of amorphous silicon (a-Si) and their mechanical flexibility can be high^15^, their limited carrier mobility (~1–10 cm^2^/Vs) may be insufficient for high-speed or low-power applications, and their chemical stability, while improving, is less than that of typical inorganic materials.

**Figure 1:**
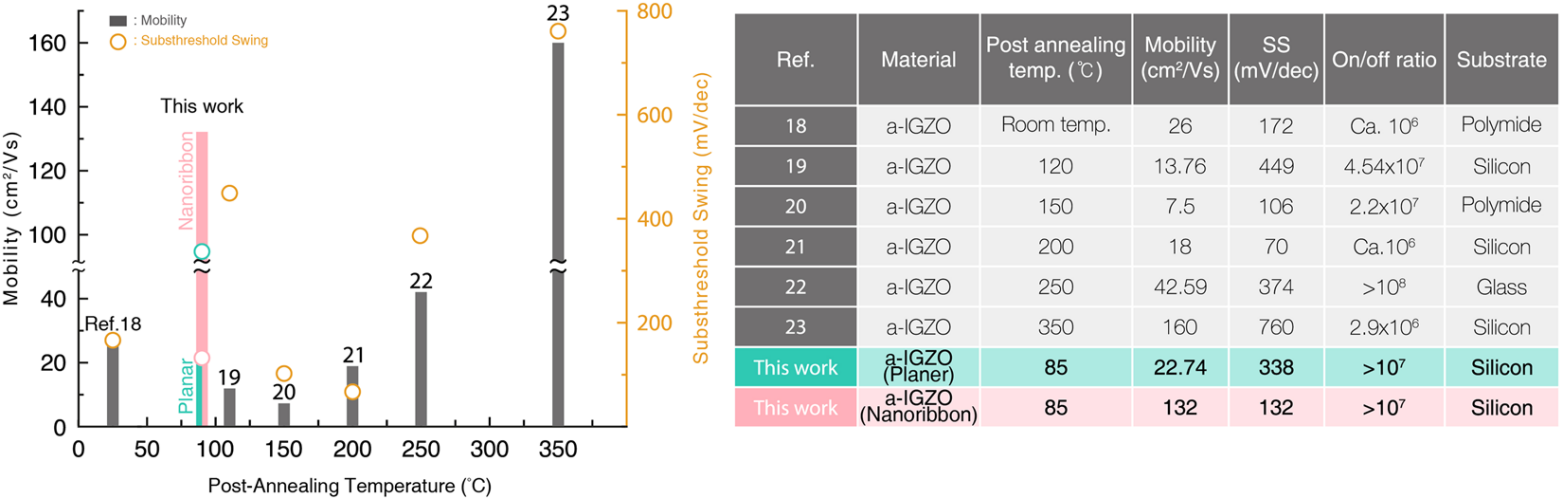
Recent progress in AOS-based thin-film transistors﻿ (TFTs)﻿. Post-annealing temperature versus mobility and Subthreshold swing (SS) achieved in recent studies into a-IGZO TFTs. A summary of detailed device specifications and substrates is shown in the table. The nanoribbon material shows the optimal combination of low temperature, high mobility, and low subthreshold slope.

Meanwhile, higher mobility (>10 cm^2^/Vs) has been obtained in inorganic amorphous oxide semiconductors (AOSs) such as amorphous indium gallium zinc oxide (a-IGZO)^16^. However, for AOSs based electronics, a trade-off exists between post-annealing process and electrical performance because of an inherent large number of defects and loose amorphous networks inside AOS films^17^. Thus, many attempts have been made, by optimizing device structure or material processing, to realize higher mobility with lower-annealing-temperature processes (Fig. 1)^18–23^. In addition, the intrinsic mechanical flexibility of AOSs macroscopic films cannot be still competitive to organic semiconductors^15^.

One-dimensional (1-D) nanowire structures based on both organic and inorganic semiconductors have displayed greater mechanical flexibility and electrical performance than do the corresponding two-dimensional films^24–26^. In this regard, nanoscale size results in a reduction in undesirable defects and dislocations incorporated into the channel. Nanowires made by a bottom-up approach, however, present challenges in assembly and lack of controllability in size for mass production^10^. In contrast, a top-down (e.g., lithographic) approach allows precise sizing, alignment, and placement, but typical large-area nanoscale patterning by e-beam lithography is highly time-consuming and cost-intensive. Moreover, adequate dry/wet etching systems of AOSs are not yet mature compared to silicon ones, and flexible substrates can be easily damaged by various chemicals or plasma systems.

Herein, we establish a top-down approach to fabricate parallel and laterally oriented nanoribbons (NRs) based on nano-imprinting using a master, low-temperature sputtering and lift-off. As a-proof-of concept, we demonstrated uniform, low-cost, large-scale a-IGZO NR arrays with different sizes without using dry/wet etch-patterning systems on the NR materials. The NRs showed outstanding mobility (132 cm^2^/Vs) and their arrays showed better optical transmittance than bare glass, even after a low post-annealing processing temperature (85 °C) obtained by microwave annealing. This technology offers a simple method to make NRs of arbitrary inorganic materials that can be deposited by sputtering or vapor deposition.

## Results and Discussion

### Fabrication of NRs based on T-shape patterns

**Figure 2:**
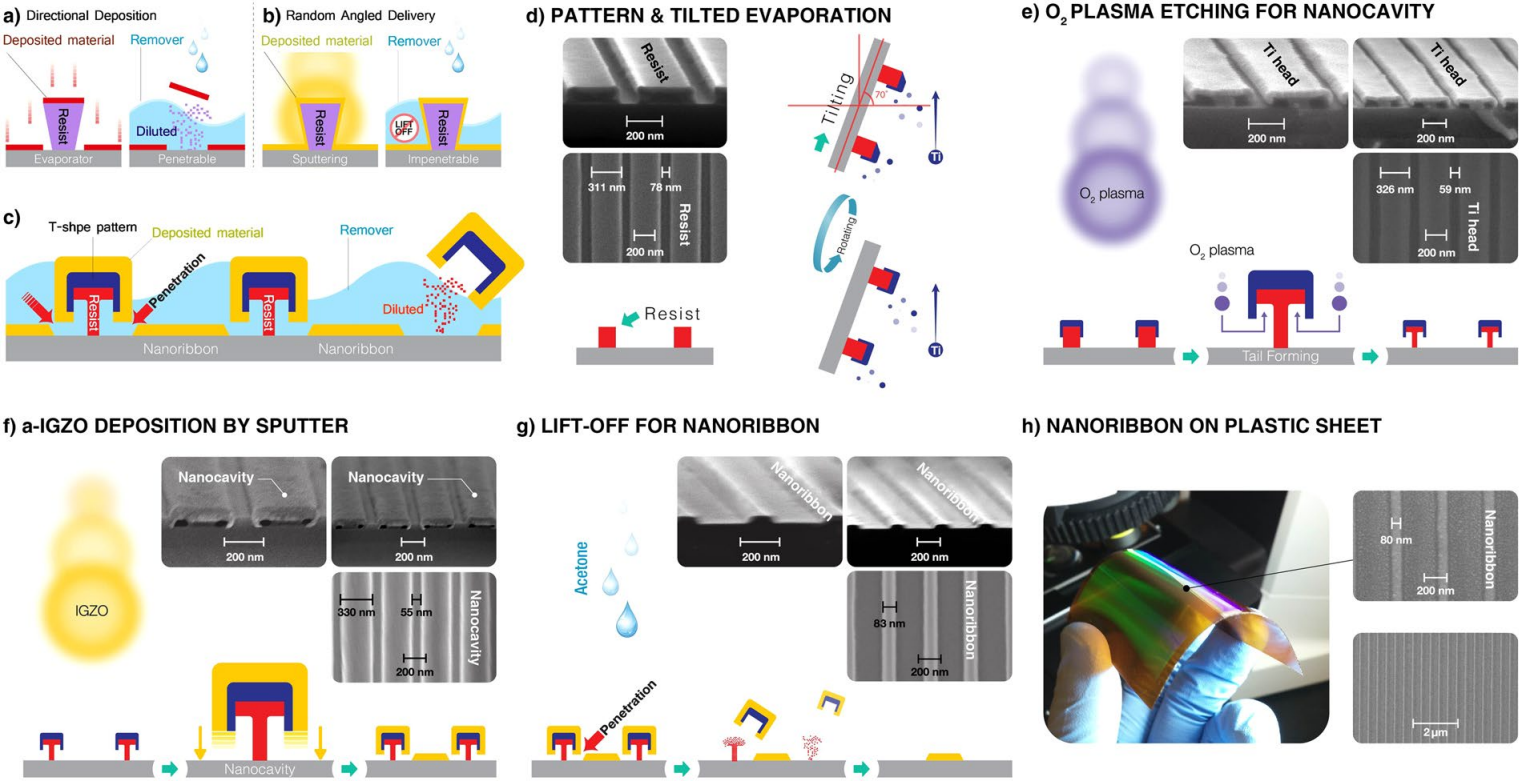
Procedures for a-IGZO NRs fabrication by lift-off. (**a**) Schematics of typical lift-off based on evaporation systems. The undercut area of the resist is not covered by directional deposition of material. (**b**) Challenges in lift-off of randomly angled sputtered films. The undercut area of the resist is covered by deposited material so solvent cannot react with the resist during the lift-off process. (**c**) The lift-off procedure based on T-shape pattern. (**d**–**g**) Cross sectional diagram and SEM images of lift-off NRs fabrication step. (**d**) Imprinted grating patterns from master stamp with 400 nm pitch, 70 nm spaces, and 120 nm height; and Ti hard mask formation by tilted depositions. (**e**) Tail creation on edge of resist by O_2_ ashing process. (**f**) a-IGZO deposition of 45 nm by sputtering on the lift-off patterns. (**g**) performing lift-off process using acetone. (**h**) Photograph and plan-view SEM images of the produced the same size of NR on PI substrate.

Typically, lift-off in device fabrication is made easier by undercut resist profiles and depositions that can be carried out directionally using evaporation sources (Fig. 2a). Unfortunately, lift-off is much less effective with sputtered films, especially for nanoscale patterning, since the sputtering systems tend to offer relatively good step coverage of undercut profiles owing to their random-angled delivery (Fig. 2b). Nevertheless, the most stable properties of AOSs are currently achieved by sputtering. By conferring a unique figure into the lift-off patterns, referred to as a “nanocavity” (under the T-shaped features of Fig. 2c), we have demonstrated a method for NR fabrication applicable to any sputtered or vapor-deposited material.

The sequence of the method is as follows: periodic grating patterns of ridges are imprinted in a resist coating silicon, glass, or polyimide (PI) substrates by a master stamp with a 70 nm space and a 400 nm pitch (Fig. 2d). The details regarding the formation of the imprinted grating pattern are depicted in Fig. S1. A 10-nm-thick titanium (Ti) cap is formed on the upper portions of the sidewalls as well as the tops of the resist ridges by tilted deposition at a 70° angle from the horizontal using an e-beam evaporator (Fig. 2d). The lower portion of the sides and the evolving underside of the resist with the Ti cap were uniformly etched using oxygen plasma, while forming the “nanocavity”, while not etching near the Ti-resist top interface (Fig. 2e), with the Ti acting as a hard mask. Sputtered atoms from IGZO target are deposited between the Ti caps (Fig. 2f) with the spaces between the caps defining the regions where NRs will remain. The caps and remaining resist ridges are lifted off using acetone under ultrasonication that can penetrate into the patterns very uniformly, removing all of the T-features but leaving NRs behind. Consequently, the NRs are very homogeneous over large areas (Fig. 2g) with a trapezoidal shape owing to some deposited atoms reaching areas under the caps during the initial stage of sputtering. Single NR width (*W*_*s*_)/thickness (*T*), and spacing (*S*) between adjacent NRs were estimated by SEM as 83/45 and 320 nm (Fig. 2g). *W*_*s*_/*T*/*S* can be controlled by modifying the grating patterns of the master stamp. A different larger NR (*W*_*s*_/*T*/*S*: 130/45/166 nm) array was made as shown in Fig. S2. Also, T-feature-patterned substrates allow any kind of sputtered materials to be lifted off uniformly and this technology is also applicable to large-area PI sheets by employing the same sequence (Fig. 2h).

### Simulation of NR and planar TFTs

**Figure 3:**
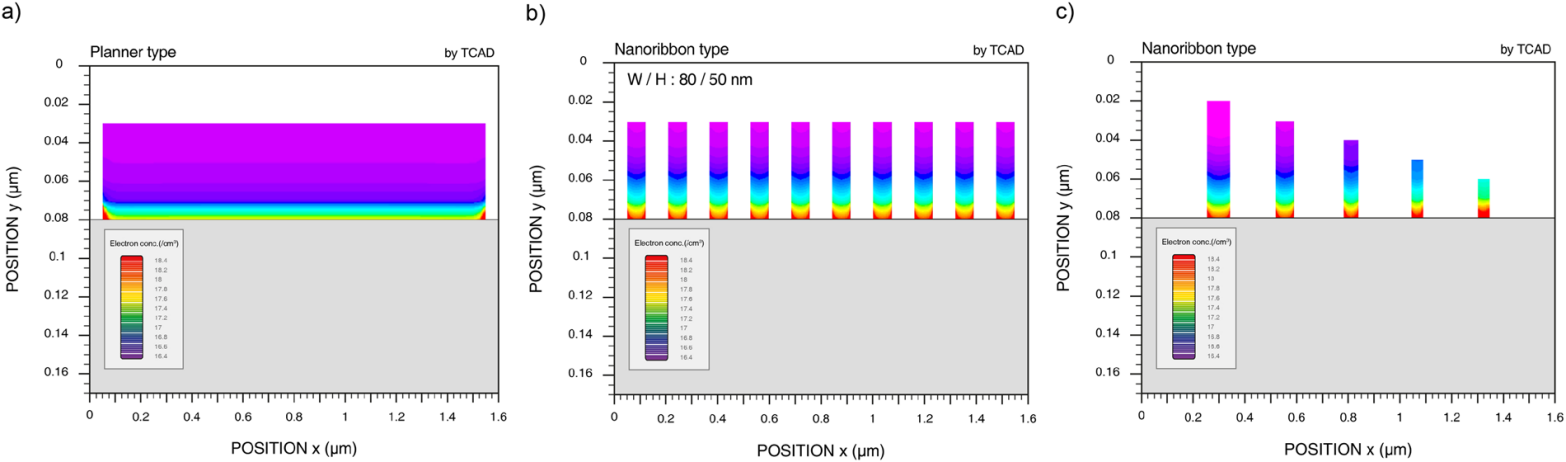
TCAD simulation of cross-sectional distribution of electron density in (**a**) planar and (**b**) NR channel with the same thickness viewed from the (source or drain) electrode. (**c**) Comparison in electron density distribution of the single NR in different sizes. Electron concentration increases as NRs shrink, as shown by the red color moving up.

We estimated cross-sectional electron distribution of a-IGZO TFTs in planar (Fig. 3a) and NR channels (Fig. 3b) with the same thickness using technology computer-aided design (TCAD) simulation. Each simulated NR TFT had 10 NRs. Higher cross-sectional electron density was obtained from NRs because of stronger electric fields confined in smaller structures and fringing field effects on the edges of the NRs^27^. We compared the electron density of NRs of different sizes in Figs 3c and S3. Electron concentrations of NRs, represented by the red color, become higher as they shrink in size. The transfer curves show higher drain current (*I*_*D*_) in larger NRs, but higher *I*_*D*_ density in smaller NRs.

### Electrical performance of NR and planar TFTs

**Figure 4:**
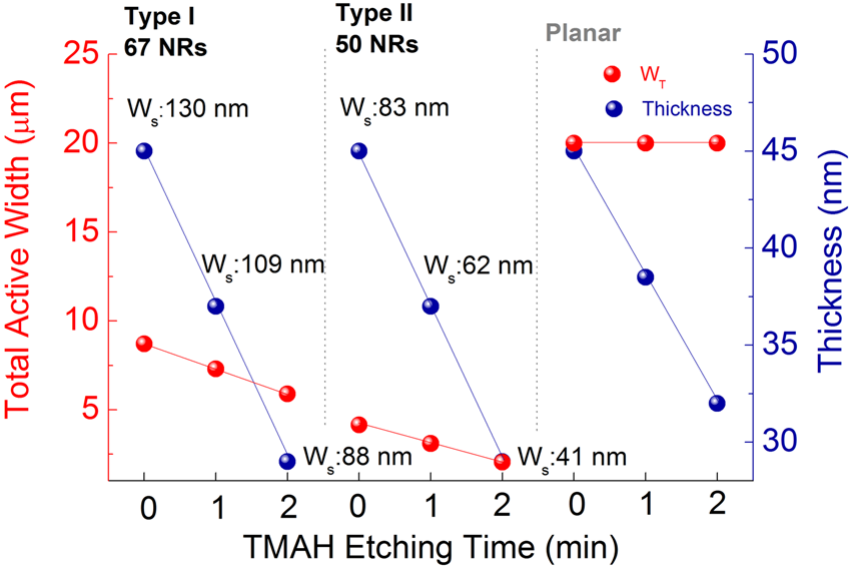
*W*_*T*_ and thickness vs TMAH etching time of each substrate. The device width and length are 20 and 10 μm, respectively. Length is fixed to 10 um for all TFTs. 67 NRs (*W*_*s*_/*T*: 130/45 nm) and 50 NRs (*W*_*s*_/*T*: 83/45 nm) are made within the device width. *W*_*T*_ was calculated by multiplying *W*_*s*_ by the number of NR for each substrate.

The electrical performance of the NRs was evaluated on Si/SiO_2_ substrates to avoid any non-ideal effects from more heterogeneous or polar dielectrics. Two different NR arrays were prepared: 67 NRs with each NR having *W*_*s*_/*T*/*S*: 130/45/166 nm (Type I) and 50 NRs with each NR having *W*_*s*_/*T*/*S*: 83/45/320 nm (Type II) within the device width (*W*_*D*_) of 20 µm. To further identify size-dependent transport properties of NRs, we etched initial NRs with tetramethyl ammonium hydroxide (TMAH) under precisely controlled conditions. Simultaneously, 45-nm-thick films were etched under the same wet etching condition as reference. TMAH etching reduced both *W*_*s*_ and *T* of the single NR at etching rates of 21.2 nm/min and 8.3 nm/min, respectively, and the thickness of planar films was etched at a rate of 6.5 nm/min. Total active width (*W*_*T*_) excluding space between NRs was estimated by multiplying each *W*_*s*_ by the number of NR within the 20 μm *W*_*D*_ as shown in Fig. 4. In the etching process of planar films, total *W*_*T*_ remained relatively unchanged while film thickness was reduced.

**Figure 5:**
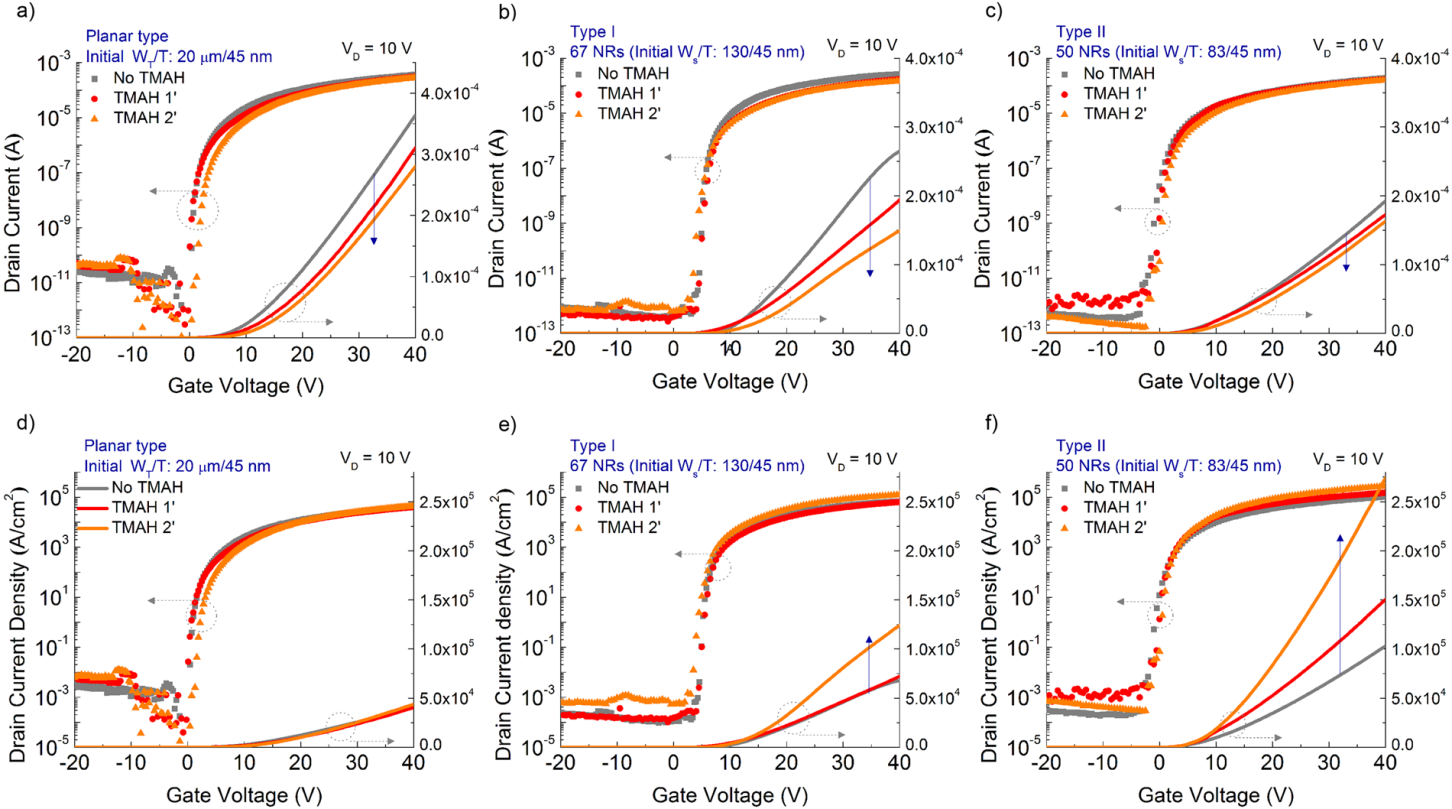
Representative transfer curves of (**a**) planar and (**b**,**c**) NR TFTs on Si substrate with respect to TMAH etching time. The Y-axis on the right side of each transfer curve shows the linear plot. *I*_*D*_ levels decreased with additional etching. Cross-sectional current density of (**d**) planar and (**e**,**f**) NR TFTs. Cross-sectional *I*_*D*_ density was estimated by dividing *I*_*D*_ by active area (*W*_*T*_ × *T*) of each channel. NR TFTs showed increased current density as NRs shrank, but the planar film TFTs did not.

Transfer curves for planar and NR TFTs under different TMAH etching times are shown in Fig. 5a,b,c. Cross-sectional *I*_*D*_ density is approximated by dividing *W*_*D*_ by cross-sectional area (*W*_*T*_ × *T*) (Fig. 5d,e,f). After etching, *I*_*D*_ levels for all TFTs are reduced because thinner or smaller sizes of channel lead to higher resistance. Cross-sectional *I*_*D*_ density, however, shows a distinguishable behavior between NRs and planar films. NR TFTs reveal higher *I*_*D*_ density with decreasing widths of NRs.

**Figure 6:**
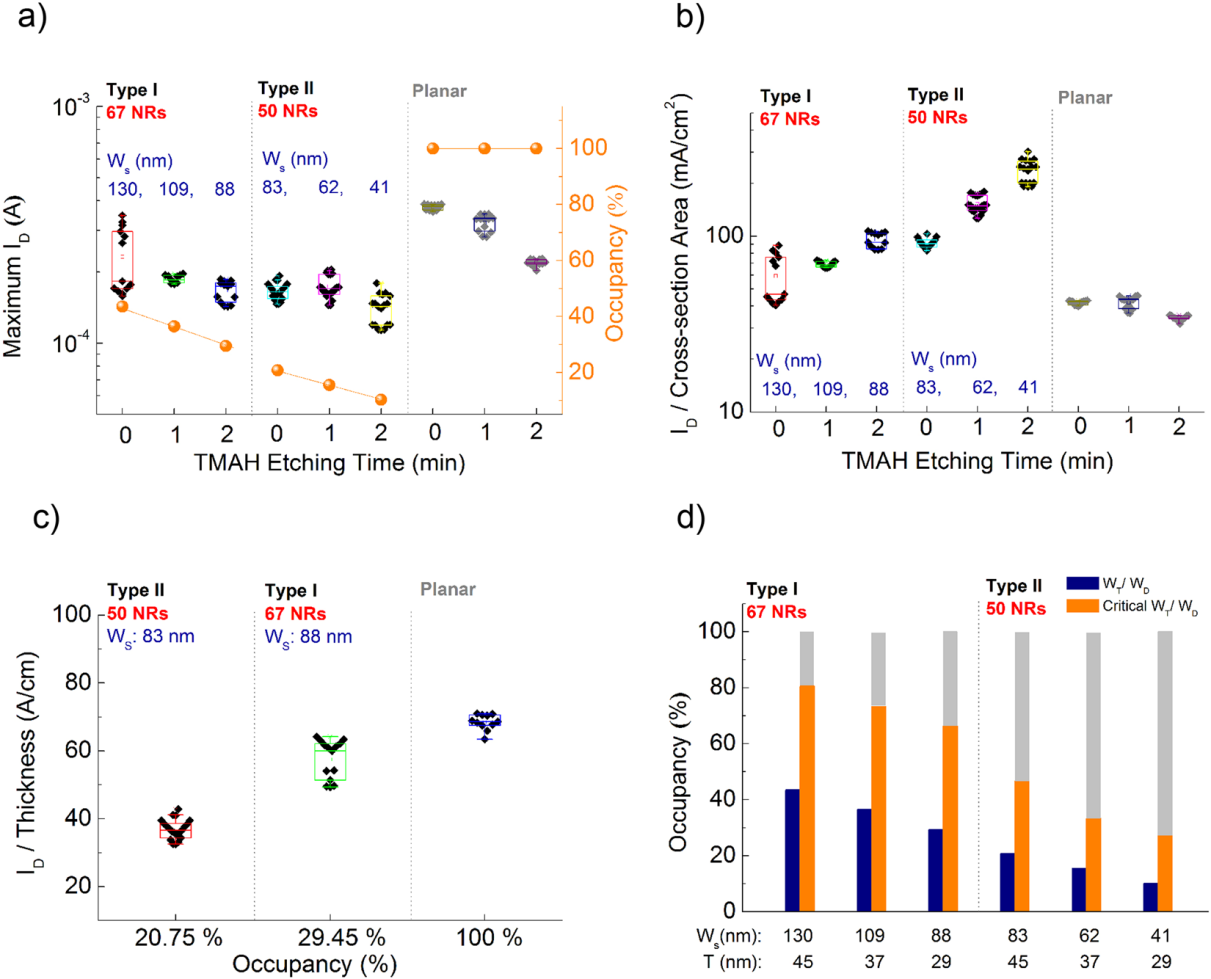
Maximum *I*_*D*_ (**a**) and *I*_*D*_ density (**b**) with respect to TMAH etching time. Maximum *I*_*D*_ was collected at *V*_*g*_ of 40 V and *V*_*D*_ of 10 V from each transfer curve. Occupancy, y-axis on the right side, was calculated by *W*_*T*_/*W*_*D*_. Cross-sectional *I*_*D*_ density was estimated by dividing maximum *I*_*D*_ by active region (*W*_*T*_ × *T*). (**c**) Correlation of *I*_*D*_ to the number of NRs. *I*_*D*_ levels from similar size NRs were normalized by each thickness for comparison. (**d**) Occupancy of NRs within the *W*_*D*_ depending on TMAH etching (blue). Critical occupancy of NRs that is required to obtain the same *I*_*D*_ level of as planar TFT is shown in orange. Higher occupancies, shown in gray, will give *I*_*D*_ greater than does the planar TFT.

For further analysis, maximum *I*_*D*_ (Fig. 6a) and *I*_*D*_ density (Fig. 6b) at 40 V gate voltage (*V*_*g*_) were collected from each transfer curve. *I*_*D*_ levels of planar TFTs are significantly dependent on etching time but the NR TFTs are relatively independent. In contrast, *I*_*D*_ density increases greatly as the size of NRs shrinks (Fig. 6b), which corresponds to the simulation result in Fig. S4. At this point, the reduced thickness in the planar film simply increased channel resistance but the decreased size of NR yielded more *I*_*D*_ from the fringing effect induced by much stronger electric fields on smaller NRs. Meanwhile, we could expect *I*_*D*_ levels from the NR TFTs to increase for denser NRs within the *W*_*D*_ (Fig. 6c) through the comparison of *I*_*D*_ levels in similar size NRs made by different master stamps. Also, we calculated critical occupancy of NRs based on the experimental results in Fig. 6a, where the same *I*_*D*_ levels as those of the planar TFTs were observed for the NR TFTs (Fig. 6d). The critical occupancy decreased in the smaller NRs from their higher *I*_*D*_ density (orange). Denser NRs over critical occupancy can make much higher *I*_*D*_ levels than the planar TFTs (gray).

**Figure 7:**
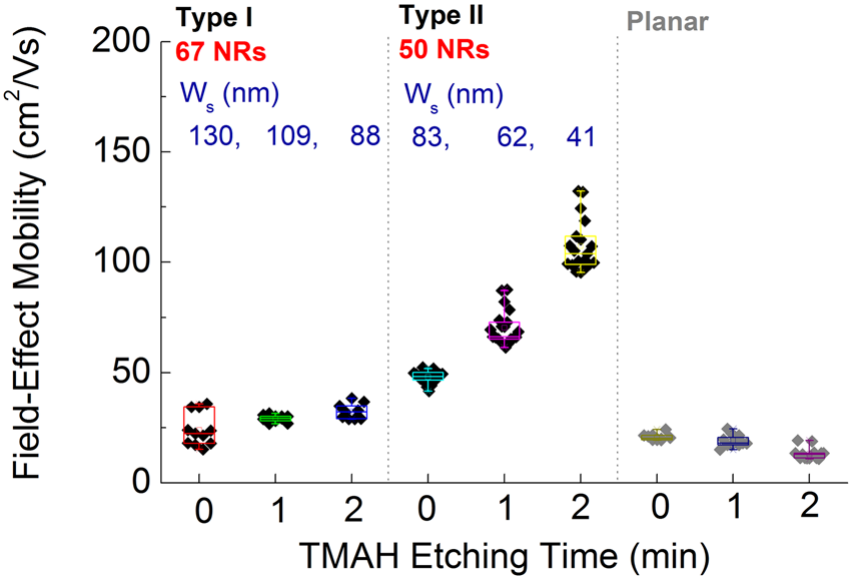
*μ*_*FE*_ vs the different sizes of NRs. Higher *μ*_*FE*_ was observed with shrinking NR.

The threshold voltage (*V*_*th*_) and on/off current ratio distribution are presented in Figs S5a and S5b. *V*_*th*_ of all devices shifted slightly to the positive regime after TMAH etching. On/off ratios of NR TFTs are over 10^7^ showing a tendency of slight decrease in on/off ratio after being etched. Effective field-effect mobility (*μ*_*FE*_)was extracted using the following equation^28^:

1 $${{\rm{\mu }}}_{{\rm{FE}}}=(\frac{{\rm{d}}{I}_{D}}{{\rm{d}}{V}_{G}})\times (\frac{L}{{W}_{T}{C}_{i}{V}_{D}})$$

where *V*_*D*_ is drain voltage (set at 10 V), *L* is the device length (fixed as 10 μm), and *C*_*i*_ is the gate capacitance per unit area. *C*_*i*_ value is experimentally obtained by a metal-insulator-semiconductor capacitor of 34.5 nF/cm^2^ (Supplementary Fig. S6). The maximum figure of d*I*_*D*_/d*V*_*G*_ was chosen in each transfer curve. *μ*_*FE*_ values of planar TFTs are slightly reduced after being etched with TMAH (Fig. 7). In contrast, *μ*_*FE*_ of NR increased up to 132 cm^2^/Vs as﻿ the size of the NRs decreased. While initial NRs made by our proposed method showed uniform electrical properties, *μ*_*FE*_ values were more disperse when NRs were made by the wet etching process.

**Figure 8:**
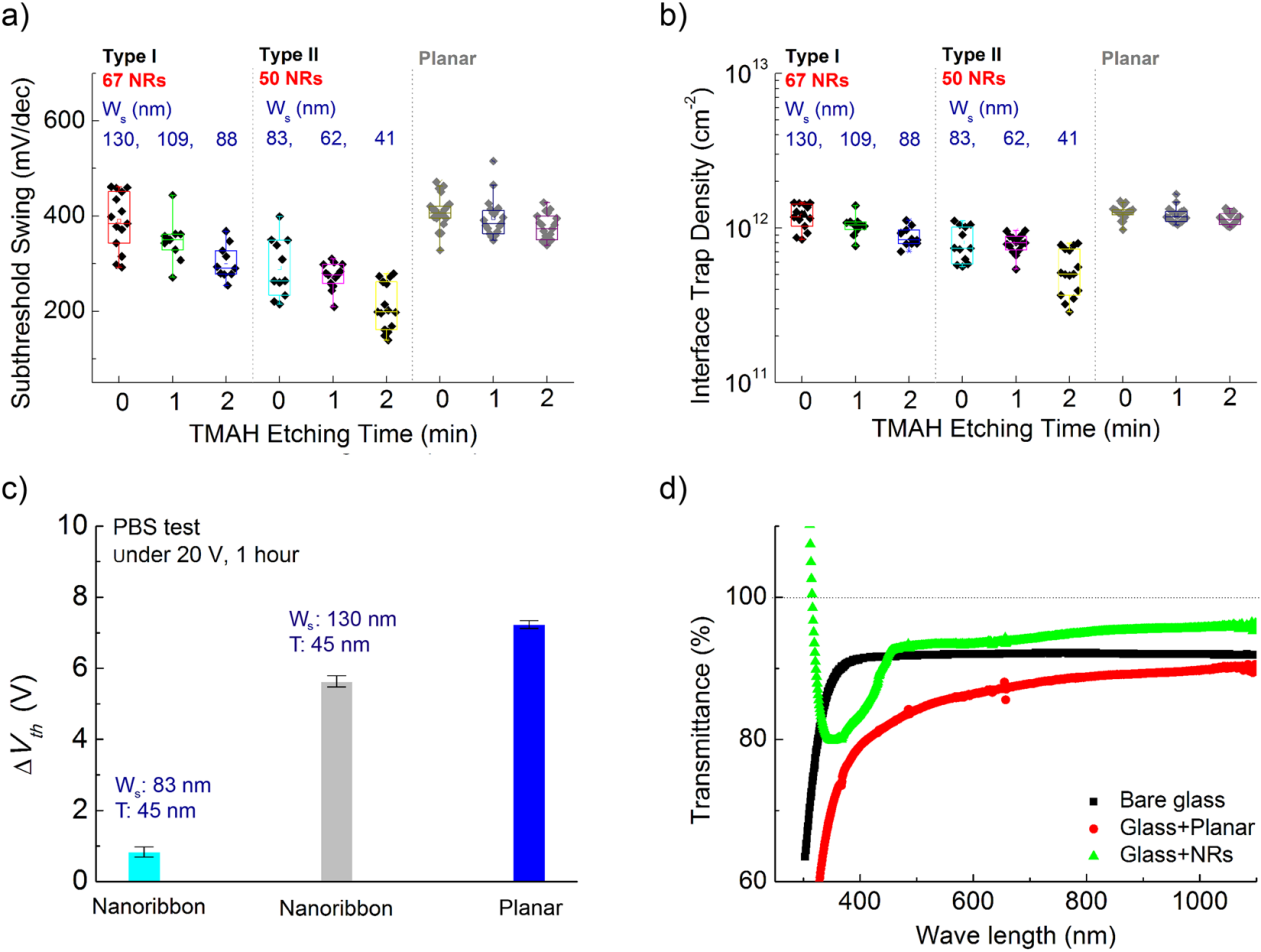
(**a**) SS and (**b**) interface trap density distribution of NR and planar TFTs. (**c**) The *V*_*th*_ shift determined by the PBS stress tests, averaged over at least 10 NRs devices and planar TFTs. The *V*_*th*_ shift is equal to the change between the initial value and the value obtained after a constant 20 V has been applied to the back gate electrode for 1 h. (**d**) Transmittance in the visible wavelength regime of bare glass, a-IGZO film, and NR structure on a glass substrate.

Corresponding to the improvement in mobility, the SS values also improved in a smaller size NR (Fig. 8a): Type II NR array shows 336 mV/dec on average for initial with best (lowest) value of 260 mV/dec and 199 mV/dec on average for TMAH 2 min with the best (lowest) value of 132 mV/dec. The planar TFTs showed a similar range of SS from 406 (initial) to 372 mV/dec (TMAH 2 min). Interface trap density (*N*_*t*_) between the channel and the gate dielectric was calculated with the following equation^29^:

2 $${N}_{t}=(\frac{SS\,\mathrm{log}(e)}{{k}_{B}T/q}-1)\frac{{C}_{i}}{q}$$

where *q* is the electron charge, *k*_*B*_ is the Boltzmann constant, and *T* is the absolute temperature. The estimated *N*_*t*_ values are lower in NRs than in planar films (Fig. 8b). Also, *N*_*t*_ is gradually lowered while the size of the NRs shrinks with less interfaces area. Furthermore, electrical stability was evaluated by positive-biased stress (PBS) tests. Electric stability was improved with less interface area; larger NR (W/T: 130/45 nm) arrays and planar type IGZO showed larger *V*_*th*_ shift under stress bias of 20 V for 1 hour (Fig. 8c). Both interface traps and the incorporation of bulk defects are highly decreased with reducing the size of NRs.

Full transparency is also recognized as a key property for flexible electronics; low loss and low reflection are both extremely desirable. By fabricating NRs and planar films on glass substrates, we were able to compare their optical transmittance, as shown in Fig. 8d; the transmittance values mentioned are relative figures, compared to air. The transmittance of the NRs reached to 92% at 550 nm that is given as greater than that of bare glass and a-IGZO/glass substrate thanks to anti-reflection effects on the surface eliminating stray light. This transmittance of our NR array is comparable to that of low dimensional materials such as grapheme (90%)^30^, carbon nanotube (92%)^31^, CuNW (84.4%)^32^, and AgNW (90.7%)^33^.

## Conclusions

A major concern for flexible electronics is to achieve high levels of device electrical performance at low processing temperatures. In this study, we investigated a promising solution, based on NR geometries. We demonstrated that a-IGZO NRs showed superior electrical performance per unit of active area and optical transparency compared to planar films of the same material, because of the structural advantages from NRs. These a-IGZO NRs are desirable building blocks for flexible electronics in wide ranging areas such as sensors, thermoelectric devices, and displays. Also, our top-down approach in fabricating NRs is widely applicable for AOSs materials based on sputtering depositions. This technology should open the door to further applications of flexible electronics based on inorganic materials.

## Methods

### Process sequence in fabricating grating pattern by nanoimprint

The silicon master containing grating pattern that is used as mother stamp is fabricated by deep ultraviolet (ASML, PAS5500/700D KRF Scanner, 248 nm) lithography and deep reactive ion etching (RIE, LAM, TCP-9400DFM). One grating pattern of the silicon master has a width of 70 nm, a space 330 nm, a height of 120 nm in a period of 400 nm. Another grating pattern of the silicon master has a width of 150 nm, a space 150 nm, a height of 120 nm in a period of 300 nm. Self-assembled monolayer (SAM, trichlorosilane Sigma-Aldrich, 97%) is coated on the silicon master template. Poly carbonate (PC) film is contacted to the silicon master, and UV resin is filled into the master pattern by roll press force to the mold. PC film mold is replicated from the silicon master by UV (365 nm) curing at an intensity of 1 kW/cm^2^ for duration of 180 sec. UV light is emitted through the PC film and cures the resist in the process. Accordingly, SAM was coated on PC film mold for better separation between the mold and imprint resin. The replica mold is contacted to each silicon, polyimide, and glass substrate with thermally sensitive resist (Poly(methyl methacrylate (PMAA), mr-I PMAA 35 k). Thermal imprinting is performed at 130 °C in air ambient for 2 hours, and after cooling down to 90 °C, the replica mold is detached from the substrate. The schematic image in the process above is provided in Supplementary Fig. 1.

### Fabrication of a-IGZO NRs TFT

A 10-nm-thick Ti cap is formed on the upper portions of the sidewalls as well as the tops of the resist ridges by tilted deposition at a 70° angle from the horizontal using an e-beam evaporator. The lower portion of the sides and the evolving underside of the resist with the Ti cap were uniformly etched using oxygen plasma using oxygen plasma at 300 W for 12 min in ambient Ar (300 ml/min). A 45-nm-thick a-IGZO was deposited by RF magnetron sputter at a power of 100 W and a working pressure of 6 mToor using Ar gas with a flow rate of 30 sccm (cm^3^/min). T-shape structures are lifted off using acetone under ultrasonication. The length and width of devices defined by photolithography that were 10 μm and 20 μm, respectively, and 50 nanoribbons were included in that width. Post deposition annealing was performed using a microwave annealing system at 1000 W for 10 min in ambient N_2_. The amorphous phase of our sputtered IGZO was maintained even after furnace annealing of ca. 400 °C^30^. In this work, the maximum internal temperature of the microwave annealing system, as detected by a thermocouple, was ca. 85 °C. Subsequently, a 10-nm-thick titanium layer and a 50-nm-thick indium tin oxide layer were deposited for the source/drain (S/D) electrode by an e-beam evaporator. S/D electrode needs to be formed in the direction perpendicular to that of NRs for the TFTs to operate (Fig. S7). A 100-nm-thick dielectric of SiO_2_ was deposited by sputtering for passivation. All electrical transport measurements were performed in air at room temperature using a shielded probe station with triaxial cable and connectors in order to minimize RF noise.

### Additional Information

Process sequence in fabricating grating pattern by nanoimprint, SEM image of nanocavity and nanoribbons using a different mother mold, simulations of different size nanoribbons, transfer curve, on-current, threshold voltage, on/off current ratio, and SS distribution of TFTs with nanoribbons and planar channel, capacitance measurement of gate dielectric, and transfer curve made by different direction of NRs.

## Electronic supplementary material

Supplementary Materials
