# Supplementary material for: Top-down Fabrication and Enhanced Active Area Electronic Characteristics of Amorphous Oxide Nanoribbons for Flexible Electronics
Source: Sci Rep. 2017 Jul 18;7:5728. doi: 10.1038/s41598-017-06040-2 (PMC5516029; doi:10.1038/s41598-017-06040-2)
Supplement: Supplementary file 1 — Supplementary Materials [file 41598_2017_6040_MOESM1_ESM.doc]

Top-down Fabrication and Enhanced Active Area Electronic Characteristics of Amorphous Oxide Nanoribbons for Flexible Electronics

Hyun-June Jang^a,b^, Ki Joong Lee^b^, Kwang-Won Jo^c^, Howard Katz^a^*, Won-Ju Cho^c^,* & Yong-Beom Shin^b^,*

^a^Department of Materials Science and Engineering, Johns Hopkins University, 3400 N. Charles St, Baltimore, USA

^b^Hazards Monitoring BioNano Research Center, Korea Research Institute of Bioscience and Biotechnology, 125 Gwahak-Ro, Yuseong-Gu, Daejeon 305-806, South Korea

^c^Department of Electronic Materials Engineering, Kwangwoon University, 20 Gwangun-ro, Nowon-gu, Seoul 139-701, South Korea


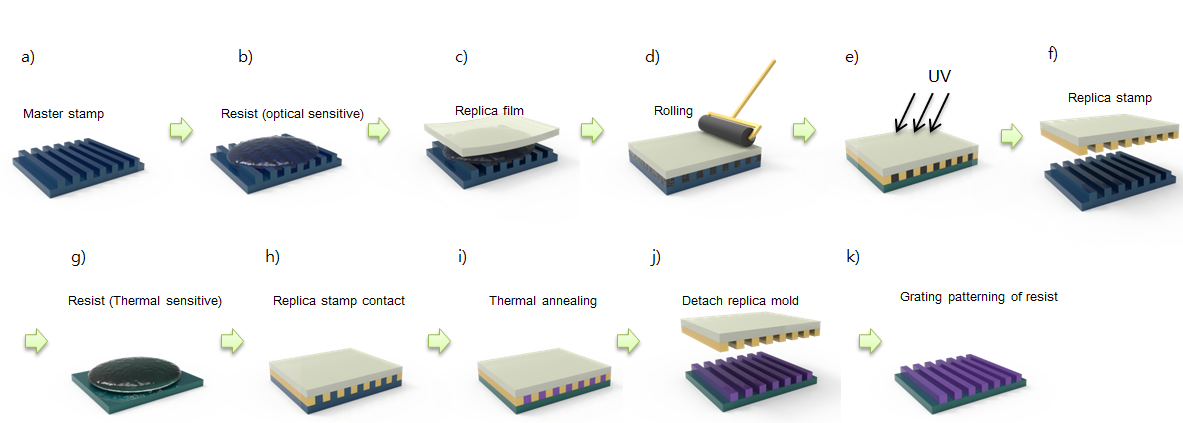


Figure S1. **Process sequence in fabricating grating pattern by nanoimprint.** **a**, the silicon master containing grating pattern that is used as mother stamp is fabricated by deep ultraviolet (ASML, PAS5500/700D KRF Scanner, 248nm) lithography and deep reactive ion etching (RIE, LAM, TCP-9400DFM). The grating pattern of the silicon master has a width of 70 nm, a space 330 nm, a height of 120 nm in a period of 400 nm. Self-assembled monolayer (SAM, trichlorosilane Sigma-Aldrich, 97%) is coated on the silicon master template. **b-d,** poly carbonate (PC) film is contacted to the silicon master, and UV resin is filled into the master pattern by roll press force to the mold. **e-f**, PC film mold is replicated from the silicon master by UV (365nm) curing at an intensity of 1kW/cm^2^ for duration of 180sec. UV light is emitted through the PC film and cures the resist in the process. Accordingly, SAM was coated on PC film mold for better separation between the mold and imprint resin. **g-h,** the replica mold is contacted to each silicon, polyimide, and glass substrate with thermally sensitive resist (Poly(methyl methacrylate (PMMA), mr-I PMAA 35k). **i-k**, Thermal imprinting is perform at 130 °C in air ambient for 2 hours, and after cooling down to 90 °C, the replica mold is detached from the substrate.


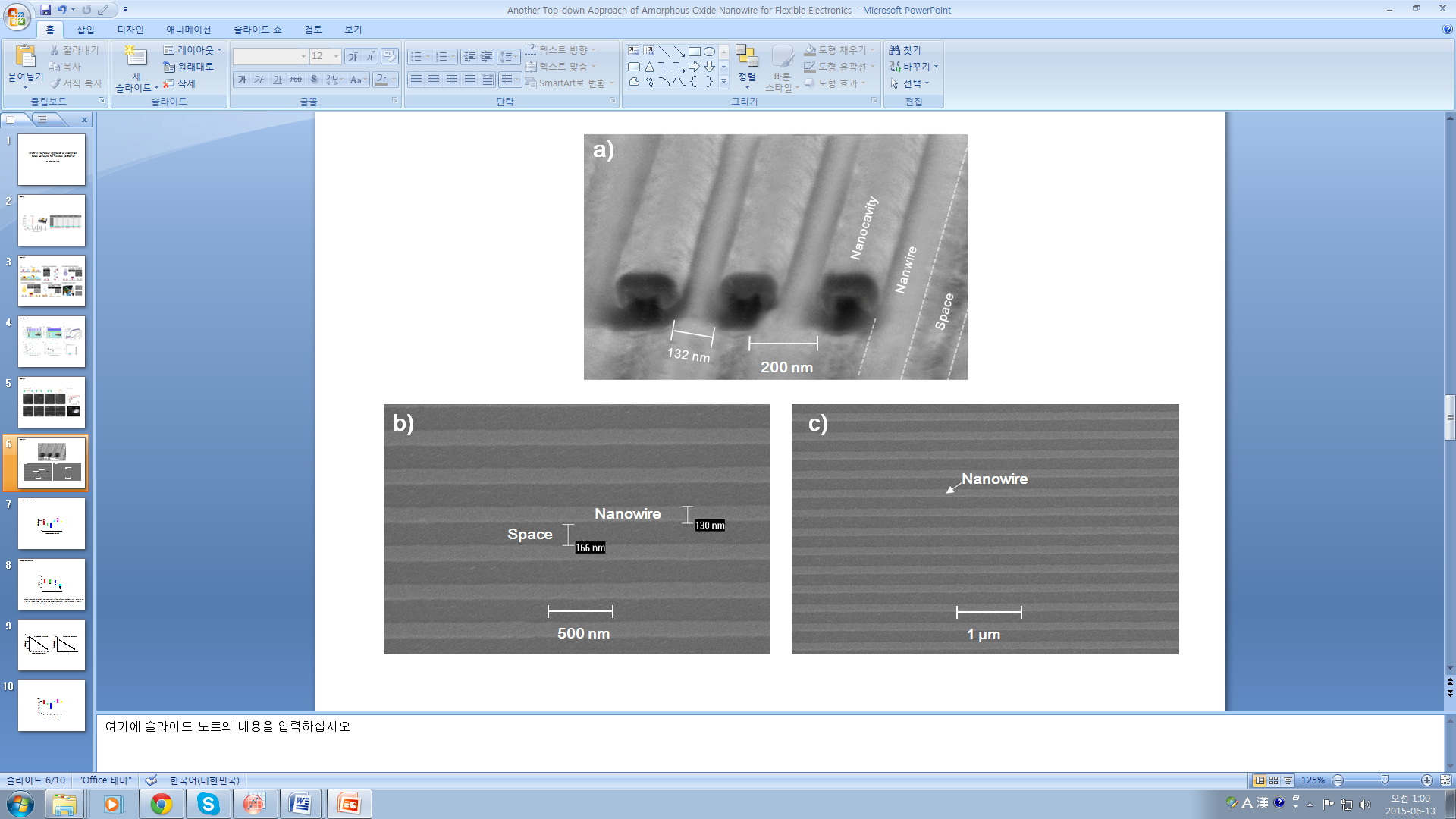


Figure S2. **a**, Cross-sectional SEM image of nanocavity when using a different mother mold with 166 nm space and 300 nm pitch. The shape of nanocavity is similar to that of Figure 2f except for their width. **b** and **c**, Plan-view SEM image of the produced nanoribbon (NR) from that mother mold. Depending on the exact layout of the mother mold, NR size or space can be controlled easily.


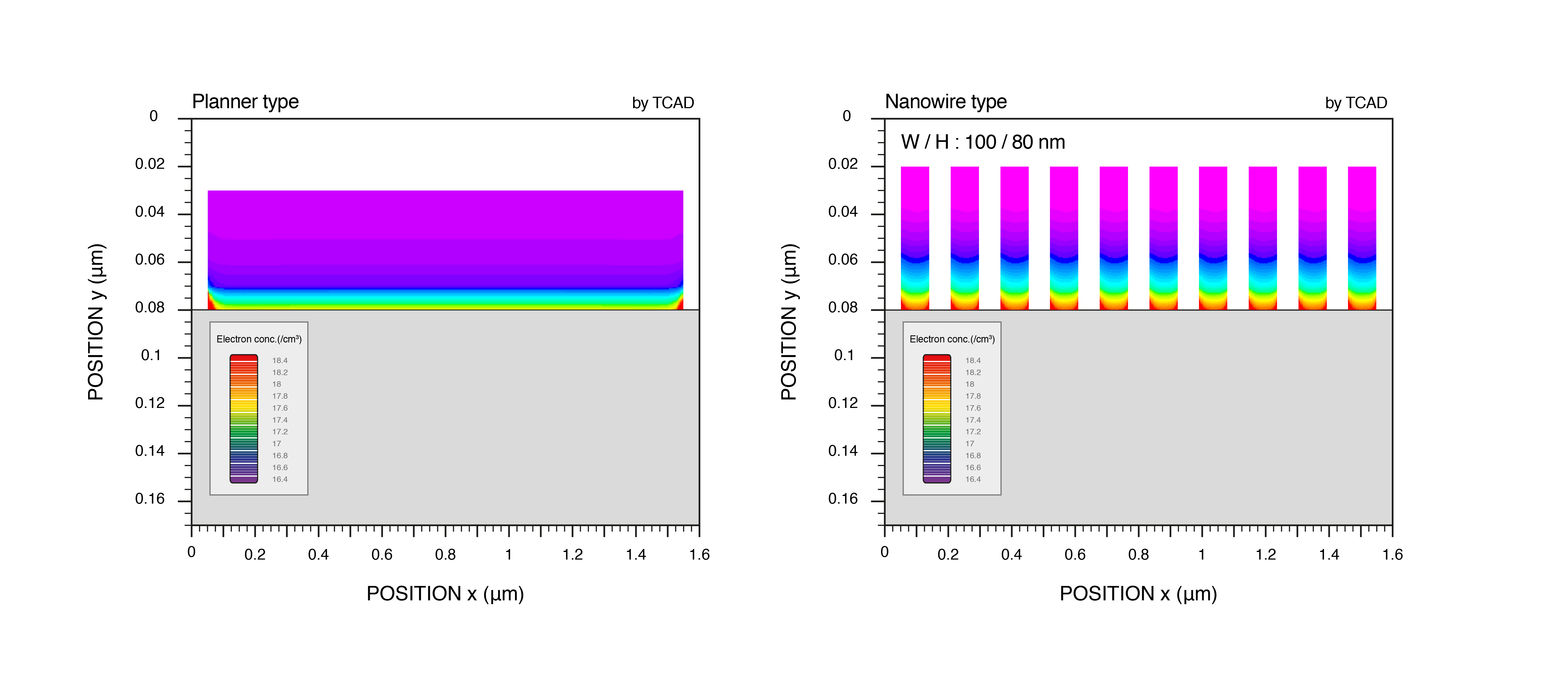

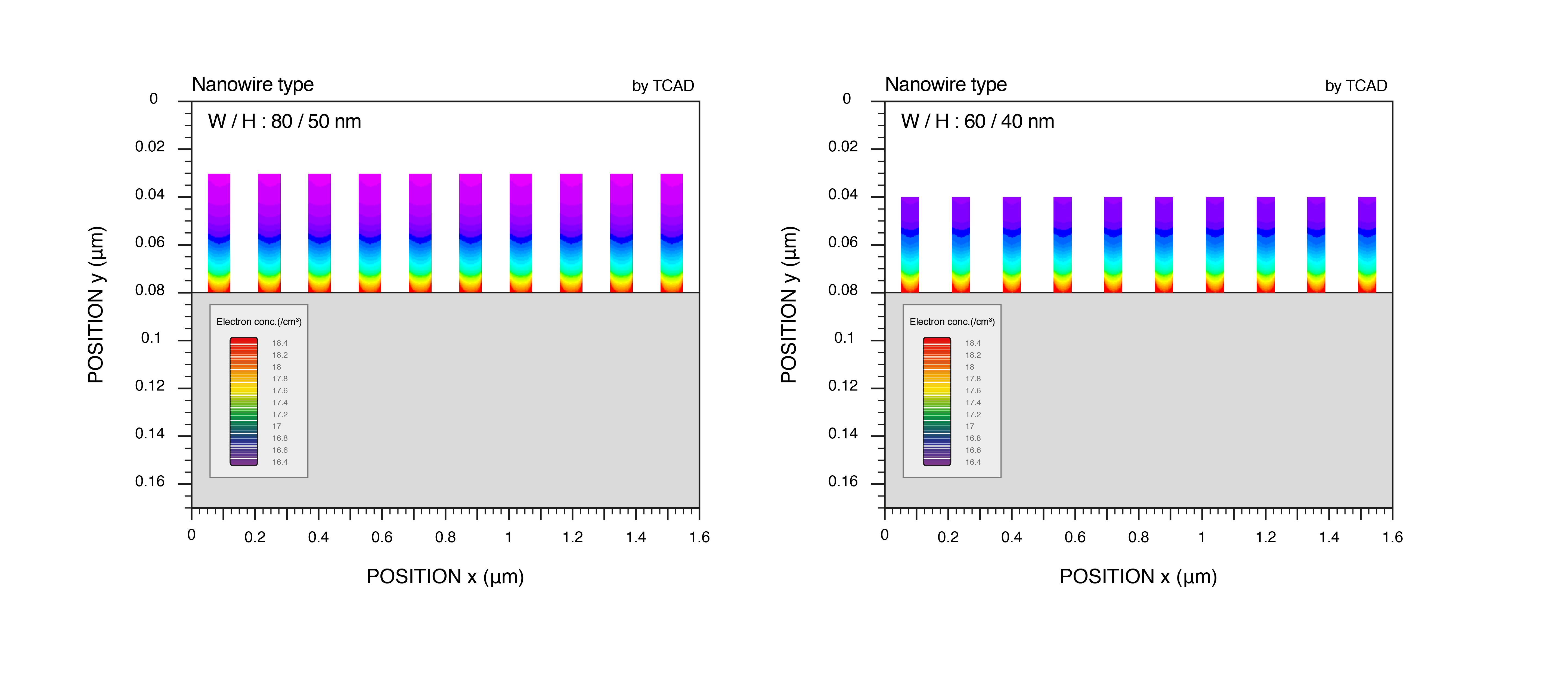


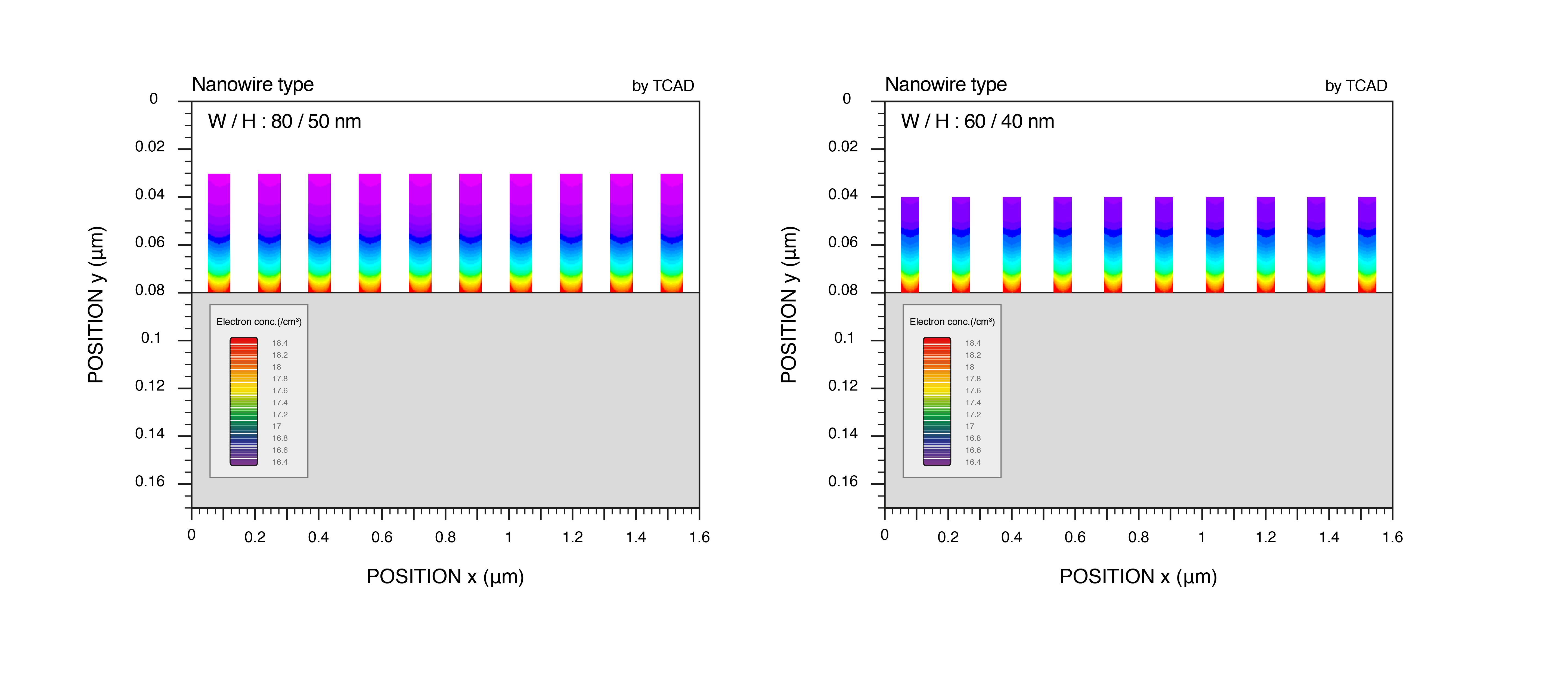

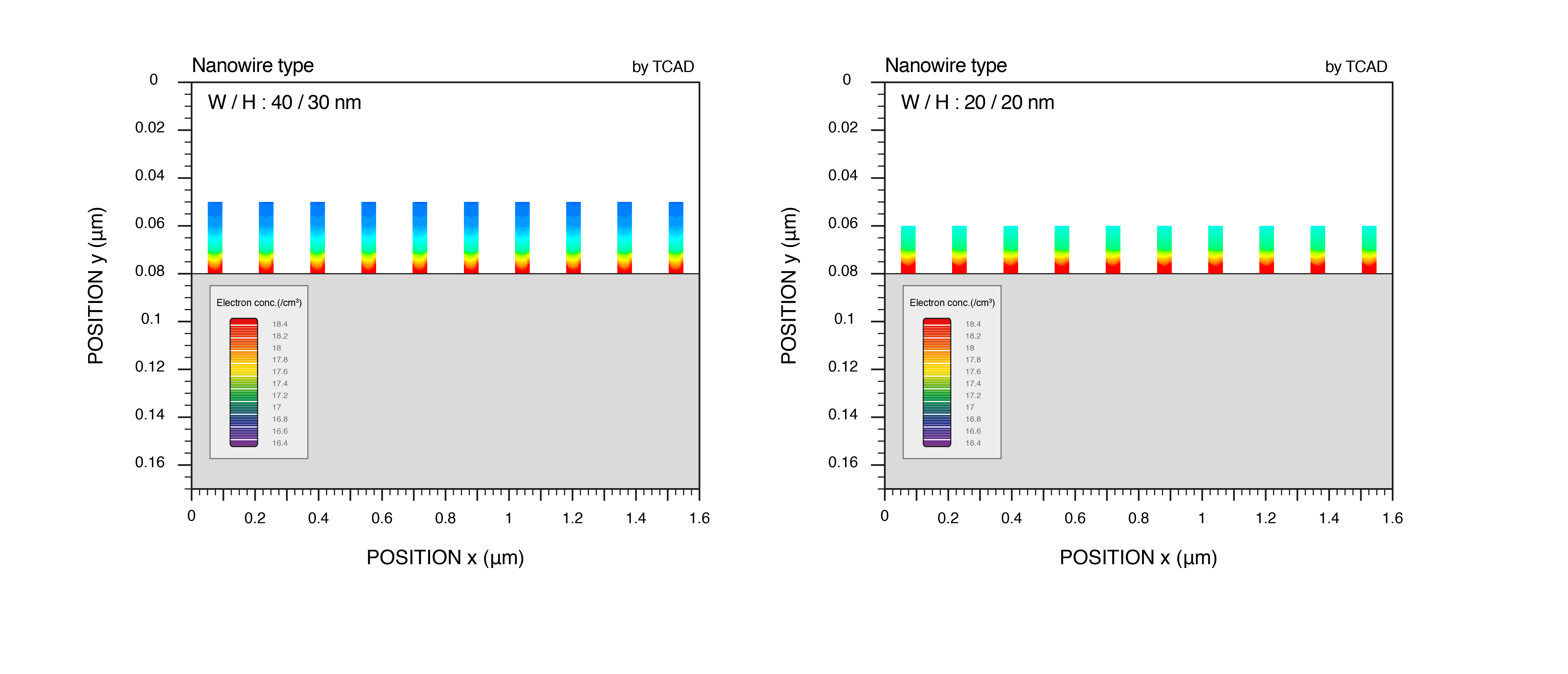


Figure S3. Cross sectional distribution of electron density in different sizes of NRs and planar channel through TCAD simulation viewed from the (source or drain) electrode. Each device has 10 nanoribbons. With decreasing sizes of nanoribbons, more high charge density current pathways are formed per unit area of oxide coverage.

Figure S4. (a) Transfer curves of TFTs are simulated based on device structures in Figure S3. The simulation result shows higher drain current (I_D_) is observed in larger NR structures but cross-sectional I_D_ density becomes higher in smaller NR structures. Current density is calculated by dividing I_D_ of each device by each cross-sectional area from 10 NRs.

**a)**

**b)**


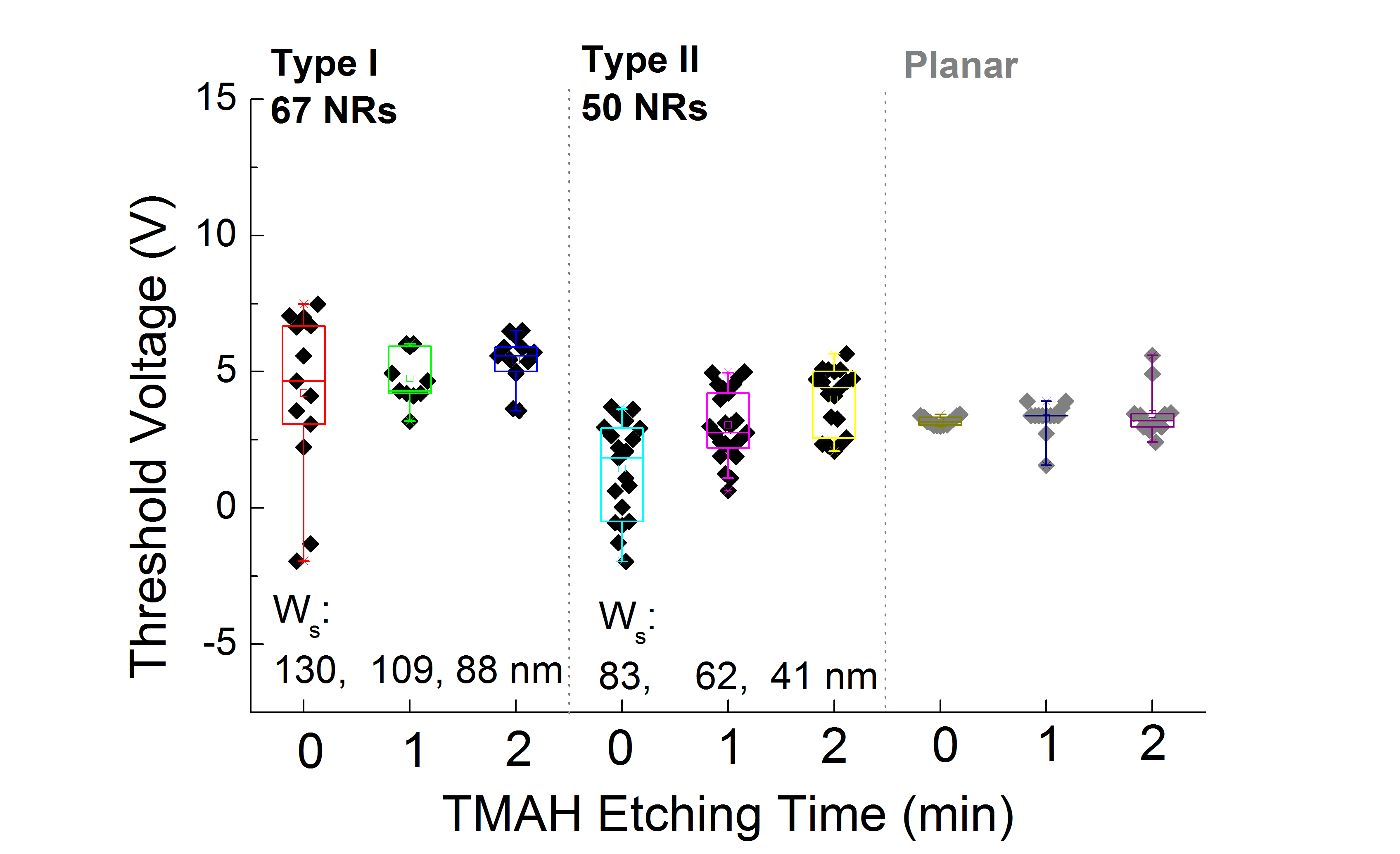

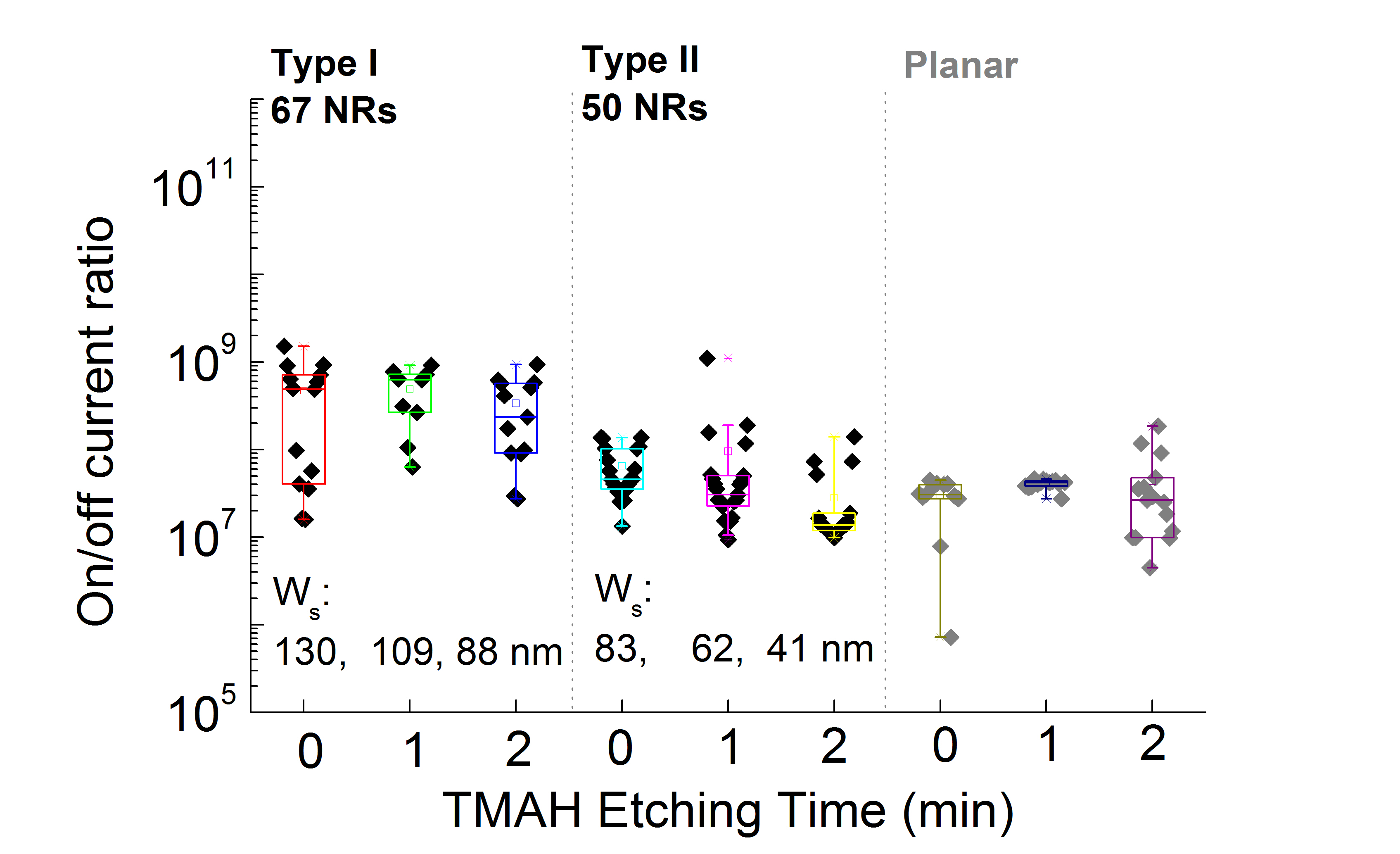


Figure S5. (a) Threshold voltage (V_th_) distribution of a-IGZO TFTs with NR and planar channel. V_th_ of all devices is slightly shifted to positive regime after TMAH etching. (b) On/off current ratio distribution of a-IGZO TFTs with NR and planar channel. On/off ratios of All NR TFTs are over 10^7^ showing tendency of slight decrease in on/off ratio after being etched. Although the planar TFT has higher I_D_ than NR counterpart, their leakage current is also higher than that of NR TFTs, which results in relatively less on-off ratio.

Figure S6. Capacitance per unit area of a 100-nm-thick SiO_2_ measured in metal insulator semiconductor structure. p-type silicon was used as substrate. A 150-nm-thick aluminum was deposited by e-beam evaporator for the metal electrode of capacitor.


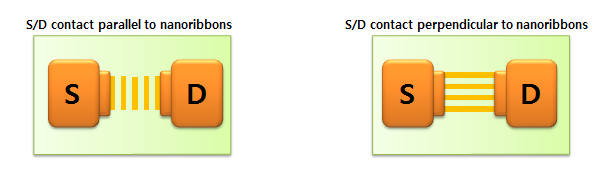


*
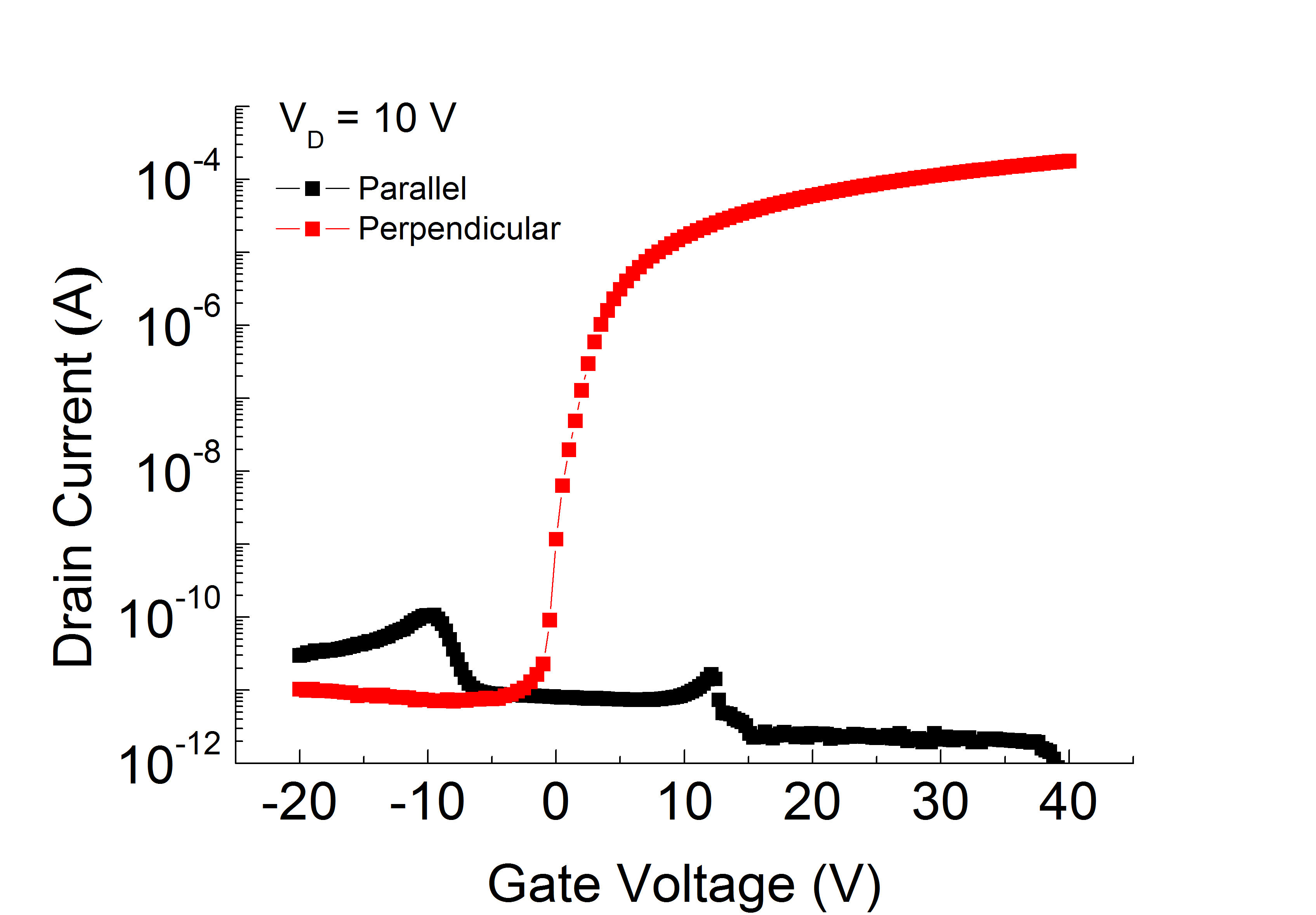
*

Figure S7. Drain current vs gate voltage depending on source and drain direction. A proper transfer curve is only obtained when the S/D formation is perpendicular to the nanoribbon.
